# Construction organoid model of ovarian endometriosis and the function of estrogen and progesterone in the model

**DOI:** 10.1038/s41598-025-90329-0

**Published:** 2025-02-24

**Authors:** Ruiqi Zhang, Yu’e Yang, Ruyue Li, Yuan Ma, Shaohan Ma, Xiuxin Chen, Bowei Li, Bei Li, XinYi Qi, Chunfang Ha

**Affiliations:** 1 Ningxia Medical University, Yinchuan, Ningxia People’s Republic of China; 2 Department of Gynecologic, General Hospital of Ningxia Medical University, Yinchuan, Ningxia People’s Republic of China; 3 Key Laboratory of Reproduction and Genetic of Ningxia Hui Autonomous Region, Key Laboratory of Fertility Preservation and Maintenance of Ningxia Medical University and Ministry of Education of China, Department of Histology and Embryology in Ningxia Medical University, Yinchuan, Ningxia People’s Republic of China

**Keywords:** Cell biology, Diseases, Molecular medicine

## Abstract

Endometriosis is a refractory estrogen-dependent gynecological disease in which ovarian endometriosis(OE) is the most common, and the main cell components are endometrial epithelial cells and stromal cells. However, constructing ectopic endometrial epithelial cell models in basic studies is still challenging. In this study, we explored the feasibility and influencing factors of constructing and validating eutopic and ectopic endometrial organoid models of OE as in-vitro models. Eutopic and ectopic endometrial tissues of OE patients were selected to establish organoids. Morphologically, the organoids showed a three-dimensional glandular structure with vacuoles or cystic irregularities, and the histological features of the epithelial organoids in endometriosis were well preserved. Immunofluorescence showed positive expression of epithelial markers and estrogen/progesterone receptors. Genetic identification revealed a 100% match between endometriosis epithelial organoids and endometrial tissue, indicating a common origin. The effects of estrogen and progesterone on the proliferation and secretion of organoids differed with the change in concentration. The successful construction of ectopic endometrial organoids provides a new in vitro model for drug intervention and mechanism study of ovarian endometriosis.

## Introduction

Endometriosis is an estrogen-dependent disease that severely impacts the quality of life and reproductive capabilities of women of childbearing age^1–3^. Approximately 80% of patients with endometriosis suffer from ovarian endometriosis (OE)^4^. Pelvic pain and infertility are common clinical features. The pathogenesis of endometriosis remains unclear, leading to limited treatment options^5^. Although pharmacological treatments and surgical interventions can help alleviate pain and address other related symptoms, the outcomes are not significant. The severity and symptoms of endometriosis vary significantly among patients, with individual differences affecting treatment outcomes^6^. Studies have shown that after surgical removal of endometrial lesions, symptoms may reappear within years, with recurrence rates as high as 27% and 58%^7^. Therefore, personalized treatment plans are necessary. Exploring the pathophysiological mechanisms and new treatment methods for endometriosis largely depends on establishing an ideal model of the disease. In previous studies, endometriosis research typically utilized two-dimensional (2D) cell lines, primary cell models, and animal models^8^. Due to phenotypic changes after multiple passages, endometriosis cell lines cannot maintain the three-dimensional (3D) structure and physiological characteristics of the original tissue. On the other hand, the proliferation of primary glandular epithelial cells from eutopic and ectopic endometrium is limited and unstable. Animal models, while costly and less efficient, also fail to replicate all features of human endometrial function^9^. Moreover, new findings in these models often do not translate effectively into human applications^8^. Constructing a reliable in vitro model is crucial for researching and treating endometriosis and improving therapeutic outcomes.

Organoid is cell models of 3D microstructures developed in vitro from normal or diseased tissues, replicating the original tissue architecture and pathological features^10^. They highly reproduce the original tissue structure and histopathological characteristics. Through long-term passage and expansion, they retain their genetic composition and tissue phenotype. To date, the emergence of various types of human-derived tumor organoids has demonstrated their ability to capture tumor heterogeneity, allowing for a more comprehensive study of disease pathogenesis^11^. Additionally, they can screen for suitable treatment options based on individual patient differences^12,13^. Organoids serve as a new research model for endometriosis^14^. In this study, we aim to construct organoid models of eutopic and ectopic endometrial epithelial cells from OE, providing an ideal model for further endometriosis study.

## Methods

### Human specimens and ethical certification

Per the Declaration of Helsinki 2000 revision’s guidelines, endometrial tissue was obtained from 24 patients with OE who received surgery at the General Hospital of Ningxia Medical University after obtaining informed consent. The ectopic endometrial tissue (ECT) was obtained during necessary laparoscopic surgery to establish ectopic endometrial organoids (ECT-O), and the eutopic endometrial tissue (EUT) was obtained during hysteroscopic biopsy to establish eutopic endometrial organoids (EUT-O). The OE patients were 25–40 years old and had regular menstrual cycles. In the previous three months, they had not received hormone drug treatment, no genital tract inflammation, and other other gynecological disorders.

Following separation, Endometrial tissue samples at proliferative stage were placed into pre-cooled 10 ml DMEM centrifuge tubes. Within two hours, the samples were transported to the laboratory. Under a laminar flow hood, fresh eutopic and ectopic endometrial specimens were processed, removing tissues with poor blood supply and excessive fibrosis. After pre-cooling with PBS, specimens were washed three times, with one piece fixed in formalin for histological staining; one piece was cryopreserved without any additives for subsequent experiments; other tissues were minced into 1-2 mm pieces using ophthalmic scissors for culturing organoids.

### Cultivation of ovarian endometrial organoids in ovarian endometriosis

Ectopic endometrial tissues from patients with chocolate cysts were minced into a paste using sterile ophthalmic scissors. To this, twice the volume of 0.1% collagenase (Sigma, Catalog C9407-100MG) and 10 μM Y27632 (a small molecule Rho kinase inhibitor, Bio-Techne, Catalog 1254) were added and mixed thoroughly. After gentle pipetting several times, the mixture was transferred to centrifuge tubes and digested at 37 °C for 1.5 h, with shaking every 15–20 min to ensure full contact and digestion. Once the digestion reached a flocculent state, AdDMEM/F12 complete medium [containing 5% fetal bovine serum (FBS; Gibco, Catalog 270–10,610), DMEM/F10 medium (Gibco, Catalog 12,634–010), 1 × GlutaMAX (Gibco, Catalog 35,050,079), and 1% antibiotic–antimycotic (Solarbio, Catalog P1400)] was added to terminate the digestion. The mixture was then centrifuged at 1000 rpm for 5 min to obtain a cell pellet, which was filtered through a 70 μm cell strainer and centrifuged again at 1000 rpm for 5 min. Matrix gel (Corning, Catalog 354234) was taken from a 4 °C refrigerator and placed on ice, then gently pipetted with the complete cell pellet to mix. The matrix gel-cell mixture was added to pre-warmed 6-well plates and incubated at 37 °C with 5% CO2 for 15 min to solidify. After the matrix gel polymerized, 2 mL of complete culture medium was added to the wells.

The complete organoid culture medium containing AdDMEM/F12 was supplemented with the following components:

**Table:** 

concentration	medium	company	Catalog
10 mM	HEPES	Gibco	15,630,080
2 mM	Glutamax	Gibco	35,050,079
80 ng/ml	EGF	PeproTech	AF-100–15
500 ng/ml	recombinant human R-Spondin-1	PeproTech	120–38
100 ng/ml	recombinant human IGF protein	Bio-Techne	291-GT
150 ng/ml	Noggin	Bio-Techne	3344-NG
150 ng/ml	Wnt3a	Bio-Techne	5036-WN
5 ng/ml	FGF-10	Bio-Techne	345-FG
10 mM	Nicotinamide	Bio-Techne	4106
1.25 mM	N-acetylcysteine	Bio-Techne	5619
8 μM	A83-01	Bio-Techne	2939
1X	B27 supplement	Life Technologies	12,587–010
1X	N2 supplement	Life Technologies	17,502–048
100 nM	glycogen synthase kinase 3 (GSK3) inhibitor CHIR-99021	MedChemExpress	252,917–06-9

### Medium exchange and passaging of endometrial epithelial organoids

The culture medium should be refreshed every three days. Once the endometrial epithelial organoids reach a size of 200 μm, they are to be passaged at a ratio of 1:2 to 1:3. For mixed and solid organoids, 2 mL of TrypLE Express (Gibco, Catalog 12,604,013) is added to each well, and the organoids are incubated at 37 °C with 5% CO2 for 10 min to facilitate digestion, with observations and mechanical shearing performed every 3–5 min. Cystic organoids can be separated solely by mechanical shearing. After digestion, the suspension is centrifuged at 1000 rpm for 5 min, and the cell pellet is mixed with matrix gel and reseeded as described above.

### Cryopreservation and resuscitation of endometrial epithelial organoids

Transfer well-grown endometrial epithelial organoids of more than 5 generations and 200 μm in size into an appropriate amount of organoid cryopreservation solution (biogenous, catalog number: E238023). Gently pipette to resuspend and immediately proceed with cryopreservation. It is recommended to cryopreserve 1 × 10^3^–1 × 10⁷ cells or the corresponding amount of organoids per 1 ml of cryopreservation solution. Place the cryovials into a gradient freezing box and store at -80 °C overnight. The next day, transfer them to a liquid nitrogen tank. For thawing: Preheat a water bath to 37 °C. Quickly thaw the cryovials in the preheated water bath while continuously shaking. After approximately 1–2 min, when the cryopreservation solution is completely dissolved, transfer the organoid cryopreservation solution into a 15 mL centrifuge tube. Add 10 times the volume of organoid passage culture buffer G (buffer volume: cryopreservation solution volume = 10:1) to resuspend, gently pipette to mix, and centrifuge at 300 g for 5 min at 4 °C. Discard the supernatant. Then, place the organoids in Matrigel, plate them, and add the culture medium.

### Histology and immunostaining

Tissue Embedding: Fix in 4% paraformaldehyde overnight, followed by dehydration and paraffin embedding. Then, section the eutopic and ectopic endometrial tissues at a thickness of 5 μm.

Organoid Embedding: Gently scrape off the organoid cultures using a pre-cooled 1000μL pipette tip with a broken end, and dissociate the organoids from the matrix gel using cell recovery solution (Corning, Catalog 354,253). Fix approximately 200–500 organoids in 4% neutral buffered formalin overnight at 4 °C. After washing, embed the organoids in a 3% agarose solution and allow them to solidify at room temperature before storing at 4 °C. Remove the agarose-solidified organoids and place them in a tissue embedding cassette for automatic dehydration, starting with gradient dehydration (75% ethanol for 30 min, 80% ethanol for 1 h, 95% ethanol for 30 min, anhydrous ethanol for 30 min twice, xylene I for 1 h, xylene II for 1 h), followed by wax infiltration (soft wax I for 30 min, soft wax II for 30 min, hard wax for 1 h), and then embedding.

For H&E staining of OE organoids, deparaffinize the paraffin sections, stain with hematoxylin for 5 min, rinse with tap water, differentiate in differentiation solution for 30 s, rinse again, soak in eosin solution for 1 min, and then rinse with tap water for 5 min. After dehydrating, clearing, and mounting the slides with neutral resin, air-dry before microscopic observation.

Periodic acid—Schiff (PAS) staining was able to show the presence of mucin and was conducted.

following the manufacturer’s instructions(Solarbio). In short, sections were dewaxed and continuously cultured in periodate solution and Schiff reagent.

Immunohistochemistry(IHC) analysis of OE organoids includes deparaffinizing the sections, antigen retrieval, cooling to room temperature, and washing three times with PBS for 5 min each. Then, block endogenous peroxidase activity for 15 min, followed by blocking with 5–10% normal goat serum for 10 min at room temperature. Without washing off the serum, add the primary antibody and incubate overnight at 4 °C. Wash the sections three times with PBS for 5 min each, apply the secondary antibody at 37 °C for 30 min, and wash again with PBS for 5 min, three times in total. Add DAB chromogen for 2 min, then thoroughly rinse with tap water. Before observation under a fluorescence microscope, perform counterstaining, dehydration, clearing, and mounting. IHC is used to assess the expression of Ki67, providing insights into the proliferative status of these organoids.

### Immunofluorescence

After antigen retrieval, paraffin sections are cooled to room temperature and washed three times with PBS containing 0.05% Tween-20 (PBST). They are then incubated with 1% BSA at room temperature for 20 min, after which the blocking solution is discarded, and the primary antibody is added and incubated overnight at 4 °C. The following day, the sections are brought to room temperature, the primary antibody is discarded, and the sections are washed three times with PBST. The secondary antibody is then added, and the sections are incubated at 37 °C for 30 min. After nuclear staining with DAPI, the slides are mounted and observed under a fluorescence microscope. Immunofluorescence detection includes EPCAM, e-cadherin, and CK7.

### Glycogen staining

Organoid paraffin sections are deparaffinized to PBS and washed three times, treated with periodic acid for 10 min followed by a 10-min rinse with tap water; Schiff’s reagent is applied for 10 min followed by a 5-min rinse; nuclei are stained with hematoxylin for 3 min (excessive staining can be differentiated with hydrochloric acid alcohol), followed by a 5-min rinse; routine dehydration, clearing, and mounting are performed.

### Transmission electron microscopy (TEM) analysis

Organoids from OE are collected, fixed in 5% glutaraldehyde at 4 °C for 2–4 h, embedded in agarose, and then fixed in 1% osmium tetroxide in the dark at room temperature for 2 h. This is followed by room-temperature dehydration, embedding, and sectioning. The sections are stained in the dark with 2% uranyl acetate saturated alcohol solution and 2.6% lead citrate solution to avoid carbon dioxide staining; ultrathin sections are observed under a transmission electron microscope, and images are collected for analysis.

### Parental origin genetic analysis

Genomic DNA is extracted from ECT-O and EUT-O using Axygen’s genomic DNA extraction kit. A 21-STR amplification scheme is used, and STR loci and the sex gene Amelogenin are detected using an ABI 3730XL genetic analyzer.

### Hormonal intervention in organoids

To determine the sensitivity of cultured organoids to estrogen and progesterone, 3–5 × 10^5 cells/matrixgel droplets are seeded in a 24-well plate and cultured for 24 h. Then, they are treated with a complete medium containing 5 μm, 10 μm, 15 μm, 20 μm estradiol (E2), and 5 μm, 10 μm, 15 μm, 20 μm progesterone for 6 days for a patient’s OE endometrial epithelial organoids, with medium change every 3 days on average. Morphological changes are observed on the sixth day of culture, and the organoids are collected and embedded for EDU staining experiments to detect changes in organoid proliferation vitality. For a patient’s P1, P3, P5 OE endometrial epithelial organoids, a 3D vitality test is conducted by seeding 1300 cells/8ul matrix gel droplet into a 384-well plate for culture, with progesterone intervention as described above. The patient’s name, progesterone concentration, organoid location, and passage number are labeled. After 6 days of intervention, the changes in organoid vitality due to hormones are detected using a 3D vitality assay kit(promega, Catalog G9681). Thaw the 3D viability assay reagent overnight at 4 °C, and equilibrate it in a water bath at 20–22℃for 30 min. Simultaneously, equilibrate the treated culture plates at room temperature (22℃) for 30 min. Add 20 μl of the 3D viability assay reagent to each well, shake on a microplate shaker for 5 min, and let it stand at room temperature in the dark for 25 min. Place the plates into the Synergy H1 microplate reader (detection code: 2,106,081) to measure luminescence values and calculate the relative viability of the organoids.

### EDU staining detection

Organoids subjected to varying concentrations of estrogen/progestogen were collected into 1.5 ml conical centrifuge tubes. To each 1 mL of complete organoid culture medium for OE organoids, 2μL of EdU storage solution (10 mM) was added after preheating. Each tube containing 500 μl of the complete culture medium for OE organoids was supplemented with an equal volume of preheated 2 × EdU incubation working solution to resuspend the organoids treated with different concentrations of estrogen and progestogen. The organoids were incubated for approximately 2 h, followed by the addition of 80% alcohol to resuspend and fix the organoids. Permeabilization solution was then added, and the organoids were incubated at room temperature for 15 min to permeabilize, followed by the addition of fluorescent click reaction solution and incubation in the dark at room temperature for 30 min. After diluting the Hoechst 33,342 staining solution for nuclear staining, confocal microscopy, and other instruments were used to detect and analyze the proportion of proliferating cells, observing cell proliferation vitality.

### Western blot

Organoids were incubated on ice for 1 h in Matrigel cell recovery solution to remove Matrigel, washed in chilled PBS, and resuspended in cold complete culture medium. Equal amounts of protein were separated by SDS-PAGE and transferred onto a nitrocellulose membrane. The membrane was blocked at room temperature for 1 h in 5% milk/TBST, followed by incubation with primary antibodies at appropriate dilutions (ER: 1:1000, PR: 1:1000, actin: 1:5000) at 4 °C overnight. After 18 h, the primary antibody was recovered, and the membrane was washed five times with 5 mL TBST. Subsequently, the appropriate secondary antibody was added, and the membrane was incubated at room temperature for 18 h. The membrane was analyzed using enhanced chemiluminescence.

### Usage of organoids

This study collected in situ and ectopic endometrial tissues from 24 patients. All samples were used to construct organoid models. Among the successfully constructed organoids, three were randomly selected for the identification of ovarian endometriosis epithelial organoids, and hormone therapy analysis was conducted on one patient.

### Statistical analysis

Statistical analysis was conducted using SPSS 26.0 (IBM, USA) and GraphPad Prism 8.0 (USA). Datas were presented as mean ± standard deviation. Differences between two groups were assessed using t-tests, while differences among multiple groups were analyzed using one-way analysis of variance (ANOVA). A p-value of < 0.05 was considered statistically significant.

## Results

### Establishment of the OE endometrial epithelial organoid model

**Fig. 1:**
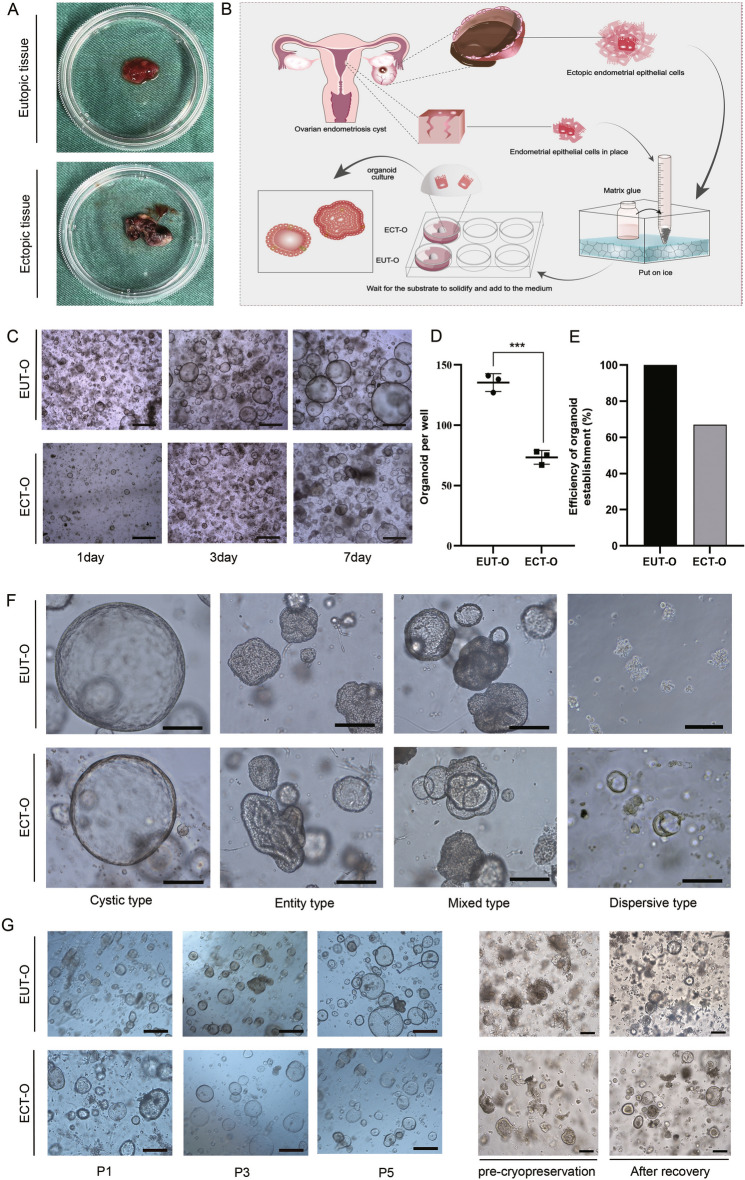
Cultivation and morphological characteristics of oe endometrial epithelial organoids (**A**) In situ endometrial and ectopic endometrial tissues from patients with ovarian-type endometriosis. (**B**) Drawn using Adobe Illustrator 2022 v26.3.1: "Workflow for culturing endometrial epithelial organoids in ovarian-type endometriosis." (**C**) Observation of EUT-O (Endometrial Epithelial Organoids) and ECT-O (Ectopic Endometrial Organoids) growth at different time points under optical microscopy (scale: 200 μm). (**D**) Proportion of EUT-O and ECT-O after 7 days of cultivation. (**E**) Success rate of EUT-O and ECT-O cultures. (**F**) Representative images depicting different morphological features of ovarian-type endometriosis endometrial epithelial organoids (scale: 50 μm). (**G**) Representative images of passaged and cryopreserved ovarian-type endometriosis endometrial epithelial organoids (scale: 100 μm).

To construct of endometrial epithelial organoid of OE, both eutopic endometrial tissue and ectopic endometrial tissue were mechanically minced and enzymatically digested (Fig. 1A). These tissues were then embedded in Matrigel for in vitro culture (Fig. 1B). EUT-O and ECT-O were successfully established,Through cultivation, we found that EUT-O and ECT-O showed certain differences on the first day after inoculation. EUT-O grew relatively quickly, in smaller quantities, and predominantly in a cystic type. In contrast, ECT-O grew more slowly, in smaller quantities, and predominantly in a mixed type(Fig. 1C). Individual endometrial cells typically formed 3D circular structures within 7 days. However, quantification of cell numbers at the same time point revealed a lower count for ECT-O compared to EUT-O (Fig. 1D). EUT-O culture was successful in all of the 24 OE patients, but ECT-O culture was successful in only 18 cases, suggesting that the success rate of ECT-O was lower than that of EUT-O (Fig. 1E). Interestingly, these two types of organoids exhibited diverse morphologies under high magnification, including dispersed, solid, cystic, and mixed types (Fig. 1F). Both EUT-O and ECT-O could be cryopreserved and revived, allowing for continuous passaging with no significant morphological changes (Fig. 1G).

## Identification of endometrial epithelial organoids in ovarian endometriosis

### Histopathological characteristics of EUT-O and ECT-O

**Fig. 2:**
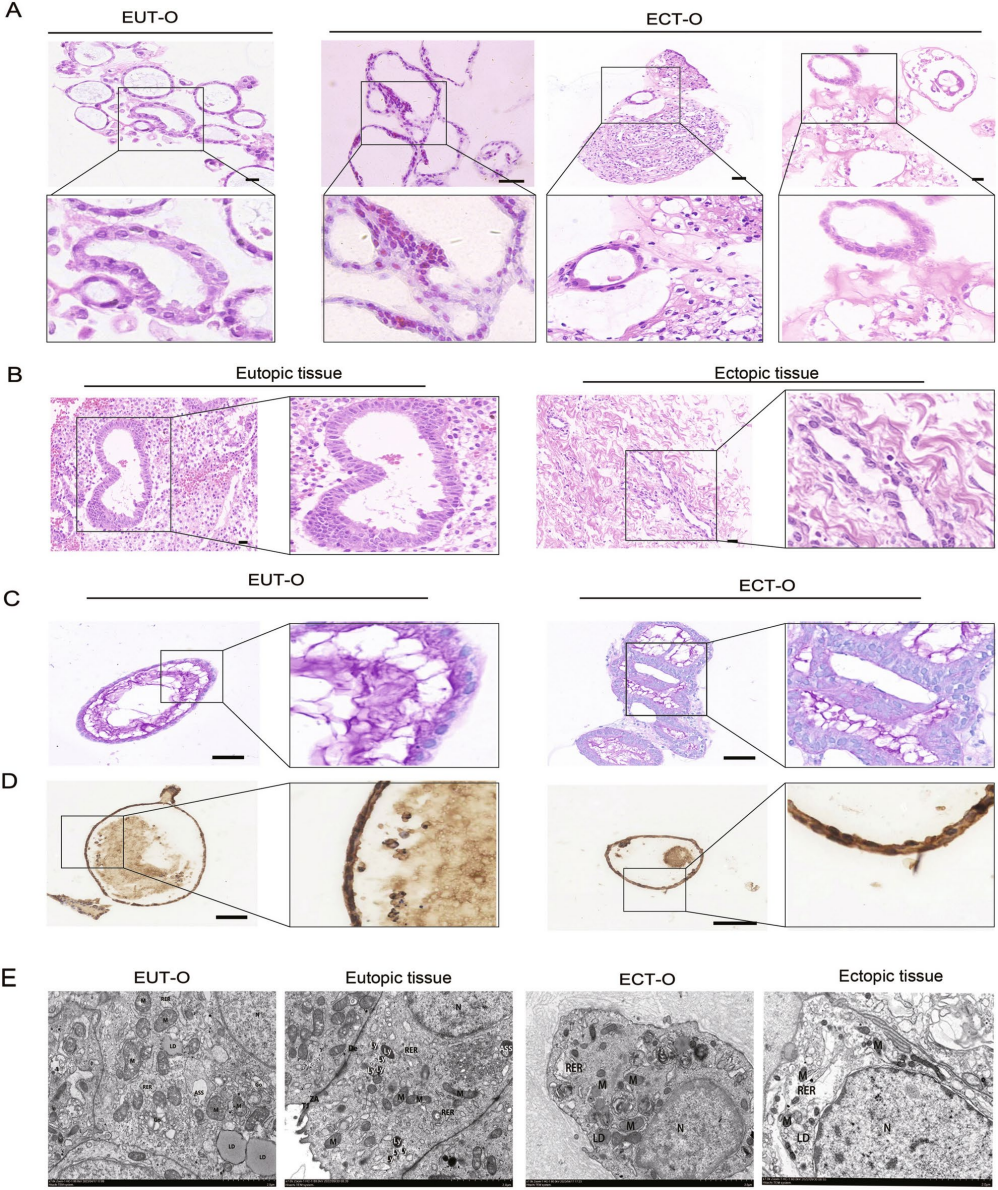
Histopathological Staining and Functional Expression of OE Endometrial Epithelial Organoids. (**A**) Hematoxylin and eosin (HE) staining of EUT-O and ECT-O (scale: 20 μm). (**B**) HE staining of in situ endometrial and ectopic endometrial tissues from patients with ovarian-type endometriosis (scale: 20 μm). (**C**) Periodic acid-Schiff (PAS) staining of EUT-O and ECT-O (scale: 20 μm). (**D**) Ki67 staining of EUT-O and ECT-O (scale: 20 μm). (**E**) Transmission electron microscopy images of EUT-O, ECT-O, and their original endometrial tissues (scale: 2.0 μm).Marked in the figure: Nucleus (N), mitochondria (M), lysosome (Ly), autophagy lysosome (ASS), rough endoplasmic reticulum (RER), lipid droplet (LD).

The endometrial epithelial organoids had recapitulated the histopathological characteristics of the parental endometrial tissue. H&E staining revealed similar pathological structures between the tissues and organoids (Fig. 2A-B). Additionally, PAS staining indicated the presence of secreted glycogen within the lumens of both EUT-O and ECT-O, suggesting that these organoids may maintain the secretory capabilities of both eutopic and ectopic endometrial glands (Fig. 2C). The expression of Ki67 in EUT-O and ECT-O confirmed the proliferative capacity of the organoid model (Fig. 2D). HE staining shows that the glandular epithelial structures of the in situ endometrial tissue are arranged neatly, with dense and regular surrounding glands. In contrast, most of the ectopic endometrial tissue exhibits sparse, disordered, and irregular glandular epithelial structures. H&E staining showed that EUT-O exhibited partially and mildly damaged glandular structures with a tendency for division at the epithelial glandular site, varied glandular lumen morphologies, and a small amount of secretion within the differently sized lumens. Typical ECT-O displayed disorganized and single or multilayered glandular epithelium, with glands formed by more cells creating separations and mostly undifferentiated lumens, irregular nuclear arrangement, and varying sizes. Some ECT-O exhibited more regularly arranged glandular epithelial cells, morphologically indistinguishable from EUT-O.

Subsequently, to further observe the ultrastructural interior of the organoids, TEM was conducted on the tissues and organoids after three generations of culture. TEM showed that EUT-O displayed a pseudostratified columnar epithelial structure similar to the parental tissue, with regular nuclear morphology, relatively uniform cytoplasmic matrix, slightly swollen mitochondria of different sizes, and essentially intact cell membranes. The rough endoplasmic reticulum(RER) and Golgi apparatus were expanded within the cytoplasm(Fig. 2E). In contrast, ECT-O only exhibited single or multilayered glandular epithelial structures, with irregular nuclear shapes, some locally concave, continuous cell membranes, uneven cytoplasmic matrix distribution, smaller mitochondria, and expanded RER in the cytoplasm (Fig. 2E). Comparing the ultrastructures of organoids, EUT-O had relatively minor overall structural damage, uniform cytoplasmic density, slightly swelling organelle, fewer lipid droplets, and lower level autophagy. On the other hand, ECT-O had relatively more severe damage, partially dissolved cytoplasm, more significantly damaged organelle, more lipid droplets, and higher level autophagy.

### EUT-O and ECT-O show characteristics of endometrium-derived epithelial cell expression

**Fig. 3:**
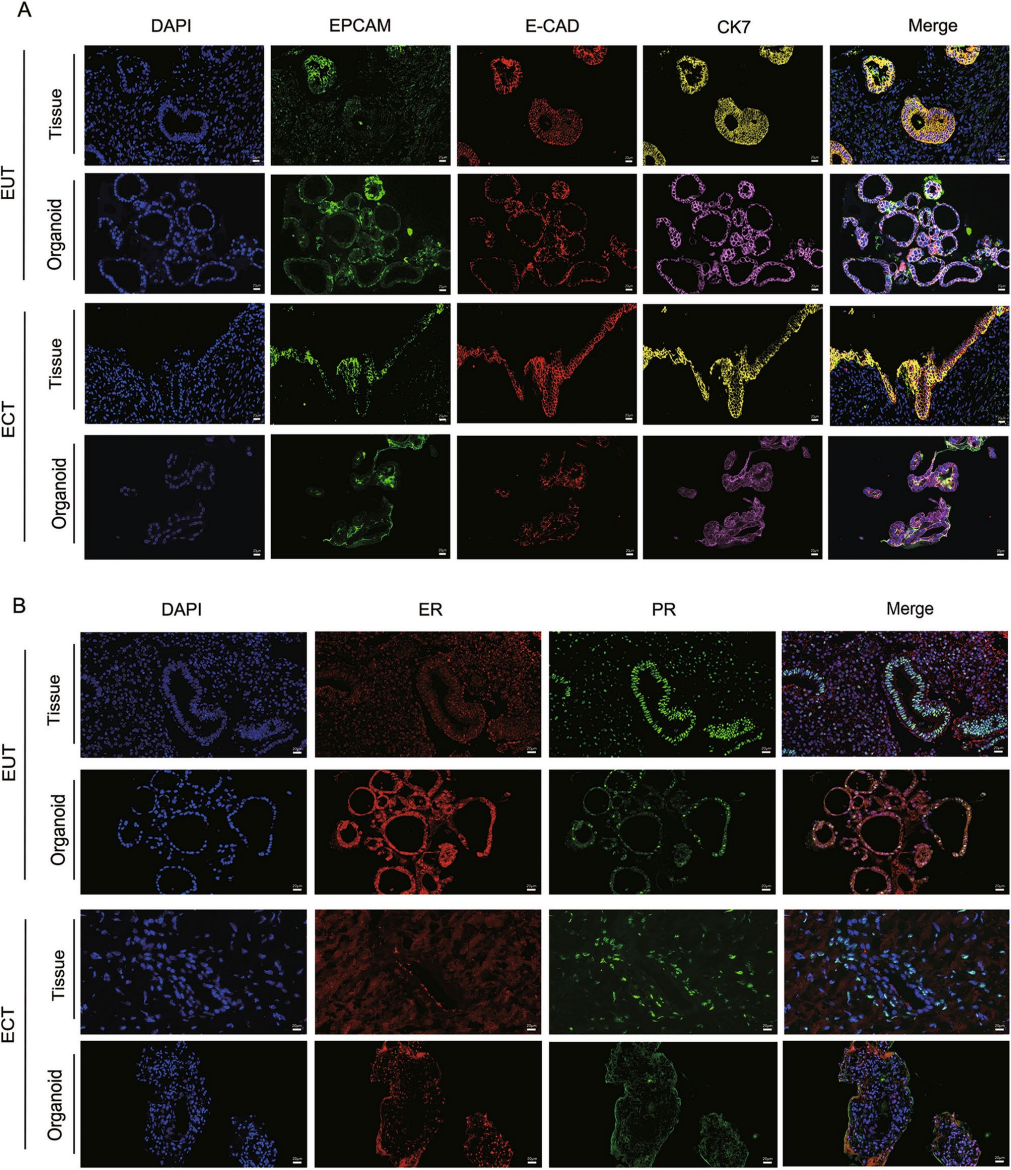
Immunofluorescence Staining for Epithelial Markers and Estrogen/Progesterone Receptors in OE Endometrial Epithelial Organoids and Original Tissues. (**A**) Expression of epithelial markers in EUT-O, ECT-O, and original tissues (scale: 20 μm). (**B**) Expression of estrogen and progesterone receptors in EUT-O, ECT-O, and original tissues (scale: 20 μm).

Immunofluorescence staining of epithelial markers (EPCAM, CK7 and E-cad), endometrial origin markers (ER and PR), and stromal markers (vimentin) for eutopic and ectopic endometrial tissues and organoids helped to confirm the endometrial epithelial origin of the cultured organoids. these biomarkers maintained good expression patterns in their corresponding organoids as in the tissues. EPCAM, CK7, E-cad, ER, and PR were stably expressed in both ECT-O (Fig. 3A) and EUT-O (Fig. 3B).

### EUT-O and ECT-O showed genetic stability with parental endometrial tissue

**Fig. 4:**
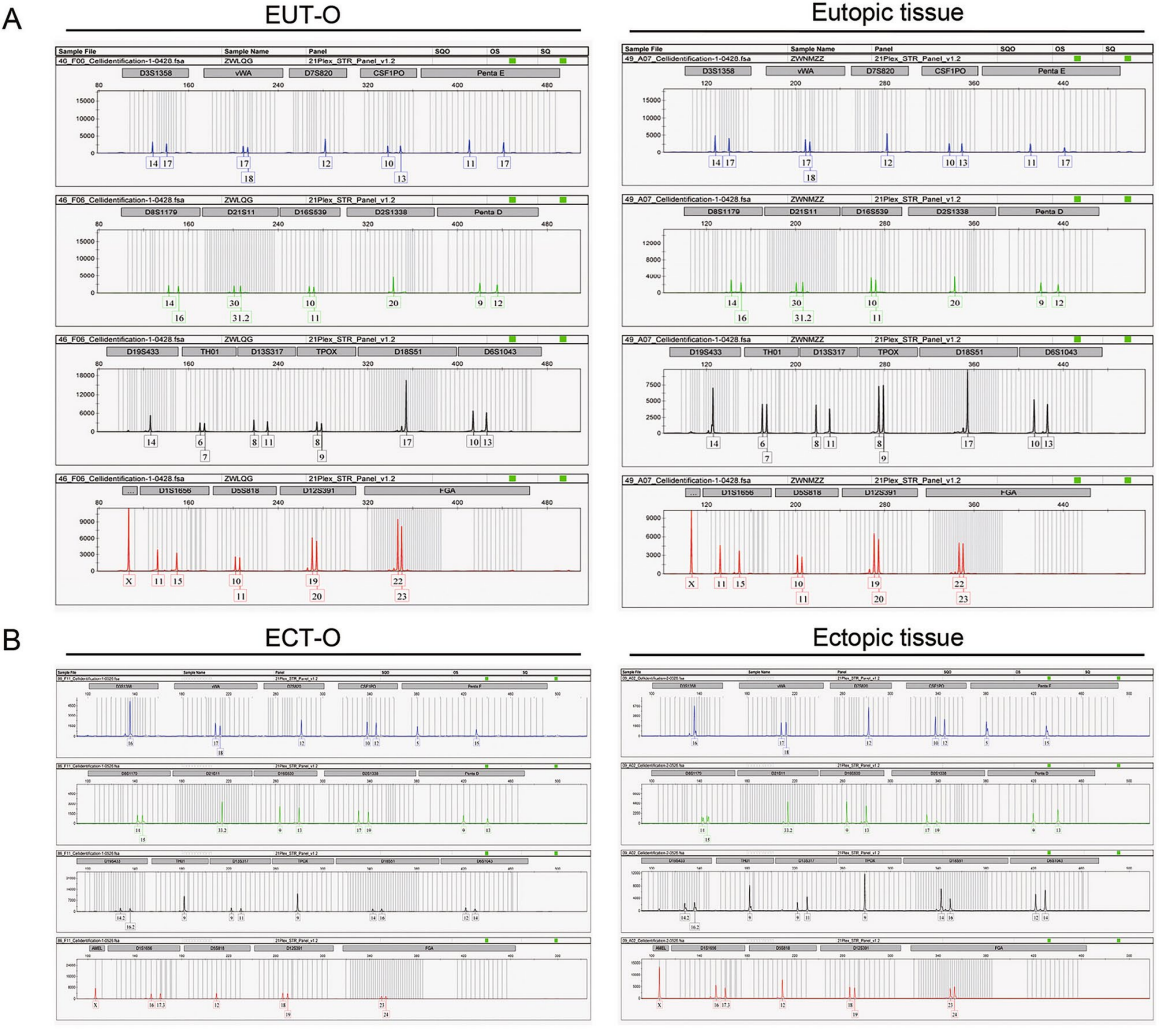
STR Profiling of OE Endometrial Epithelial Organoids and Their Source Tissues.(**A**) Genetic relatedness identification between EUT-O and in situ endometrial tissues from their source. (**B**) Genetic relatedness identification between ECT-O and ectopic endometrial tissues from their source.

After three passages of culture, the established organoids and patient tissues were compared using donor-specific gene identification. The results showed 100% match between the established organoids and the patients’ endometrial tissue, with no multi-allelic genes present. The cell typing results were satisfactory, with no significant DNA copy number abnormalities detected (Fig. 4, Table 1 and table2).

**Table 1:** Comparison of EUT-O and EUT short tandem repeat (STR) sequences.

Marker	Organoids		Eutopic endometrial tissue	
21	Allele1	Allele2	Allele1	Allele2
D5S818	10	11	10	11
D13S317	8	11	8	11
D7S820	12	12	12	12
D16S539	10	11	10	11
VWA	17	18	17	18
TH01	6	7	6	7
AMEL	X	X	X	X
TPOX	8	9	8	9
CSF1PO	10	13	10	13
D12S391	19	20	19	20
FGA	22	23	22	23
D2S1338	20	20	20	20
D21S11	30	31.2	30	31.2
D18S51	17	17	17	17
D8S1179	14	16	14	16
D3S1358	14	17	14	17
D6S1043	10	13	10	13
PENTAE	11	17	11	17
D19S433	14	14	14	14
PENTAD	9	12	9	12
D1S1656	11	15	11	15
EV值 = 1.0				

Note: Genotyping results for STR loci and Amelogenin locus in OE endometrial epithelial organoids and their source tissues.

**Table 2:** Comparison of ECT-O and ECT short tandem repeat (STR) sequences.

Marker	Organoids		Etopic endometrial tissue	
21	Allele1	Allele2	Allele1	Allele2
D5S818	12	12	12	12
D13S317	9	11	9	11
D7S820	12	12	12	12
D16S539	9	13	9	13
VWA	17	18	17	18
TH01	9	9	9	9
AMEL	X	X	X	X
TPOX	9	9	9	9
CSF1PO	10	12	10	12
D12S391	18	19	18	19
FGA	23	24	23	24
D2S1338	17	19	17	19
D21S11	33.2	33.2	33.2	33.2
D8S1179	14	15	14	15
D3S1358	16	16	16	16
D6S1043	12	14	12	14
PENTAE	5	15	5	15
D19S433	14.2	16.2	14.2	16.2
PENTAD	9	13	9	13
D1S1656	16	17.3	16	17.3
EV值 = 1.0				

Note: Genotyping results for STR loci and Amelogenin locus in ovarian-type endometriosis ectopic endometrial organoids and their source tissues.

In summary, the EUT-O and ECT-O were highly replicated the structure, physiological characteristics, and genetic features of the source tissue, constituting a reliable in vitro epithelial cell model. These organoids could be passaged, cryopreserved, and revived, making them excellent in vitro models for the study of the pathogenesis of endometriosis, drug development, and personalized medication guidance.

## Effect of gradient estrogen on the construction and functional changes of ectopic endometrial organoids in ovarian endometriosis

**Fig. 5:**
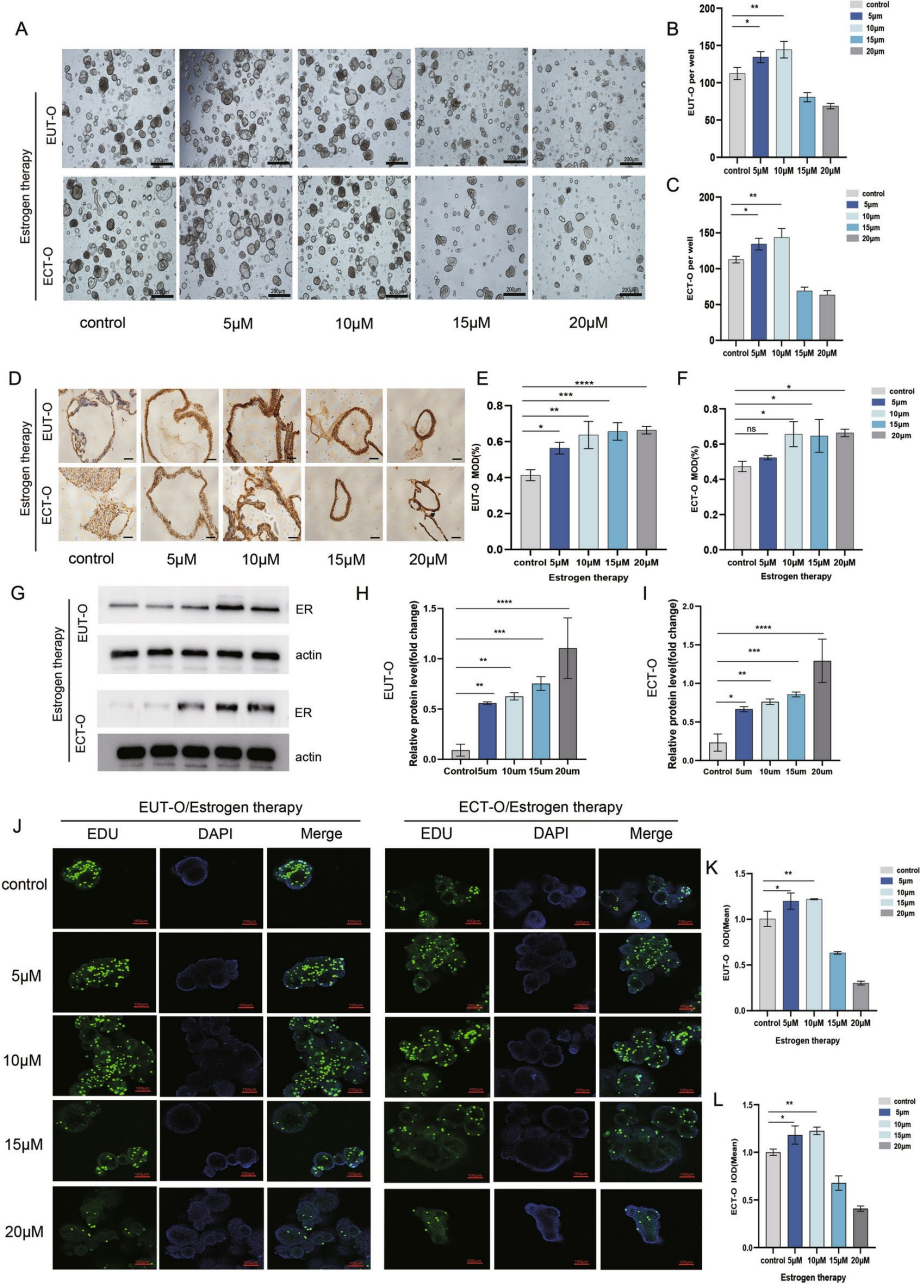
Morphological and Functional Changes in OE Endometrial Epithelial Organoids under Estrogen Intervention. Optical microscopy observation of EUT-O and ECT-O growth after 7 days of estrogen intervention (scale: 200 μm).(**A**) Optical microscopy observation of EUT-O and ECT-O growth after 7 days of estrogen intervention (scale: 200 μm).(**B**) Proportion of EUT-O cells after 7 days of estrogen intervention.(**C**) Proportion of ECT-O cells after 7 days of estrogen intervention., (**D**) Estrogen receptor expression in EUT-O and ECT-O after estrogen intervention (scale: 20 μm). (**E**) Quantitative analysis of estrogen receptor expression in EUT-O after estrogen intervention. (**F**) Quantitative analysis of estrogen receptor expression in ECT-O after estrogen intervention. (**G**) Western blot was used to detect the protein levels of ER in different groups:control , ER(5 μM), ER(10 μM), ER(15 μM), ER(20 μM). (**H**) Western blot was used to detect the protein levels of ER in different EUT-O:control , ER(5 μM), ER(10 μM), ER(15 μM), ER(20 μM). (**I**) Western blot was used to detect the protein levels of ER in different ECT-O:control , ER(5 μM), ER(10 μM), ER(15 μM), ER(20 μM). (**J**) Laser confocal images of EDU-labeled proliferating EUT-O and ECT-O under estrogen intervention (scale: 100 μm).(**K**) Immunofluorescence quantification of EDU-labeled proliferation in EUT-O after estrogen intervention. (**L**) Immunofluorescence quantification of EDU-labeled proliferation in ECT-O after estrogen intervention.

This study also aimed to observe the response of endometrial epithelial organoids to estrogen to understand the effects of hormones on organoid development.Specifically, different concentrations of estradiol (5 μM, 10 μM, 15 μM, and 20 μM) were used to intervene with two types of organoids. Microscopic observation revealed significant increase of organoids number following estrogen intervention at 5-10 μM (p < 0.05) (Fig. 5A). Interestingly, as estrogen concentration increased beyond 10 μM, the OE organoids multiplication was inhibited (Fig. 5B,C). IHC result(Fig. 5D) demonstrated that ER expression in the organoids increased with rising estrogen concentrations (Fig. 5E,F). WB results further verified that ER expression in organoids increased with the increase of estrogen concentration (p < 0.05) (Fig5G,H,I) To assess proliferation, the researchers employed laser confocal microscopy with EDU staining. OE organoids cultured with 5-10 μM estrogen showed the most pronounced proliferation tendency compared to the control group (p < 0.05) (Fig. 5J). Further validation using a 3D viability assay confirmed the proliferative status of OE organoids (Fig. 5K,L). Notably, the optimal proliferation activity was observed in the 5-10 μM estrogen cultured organoids for both organoids types, enhancing modeling efficiency. Intriguingly, EUT-O exhibited greater sensitivity to estrogen compared to ECT-O, suggesting that moderate estrogen levels promote the growth and maturation of OE organoids, facilitating their construction.

## Effect of gradient progesterone on the construction and functional changes of ectopic endometrial organoids in ovarian endometriosis

**Fig. 6:**
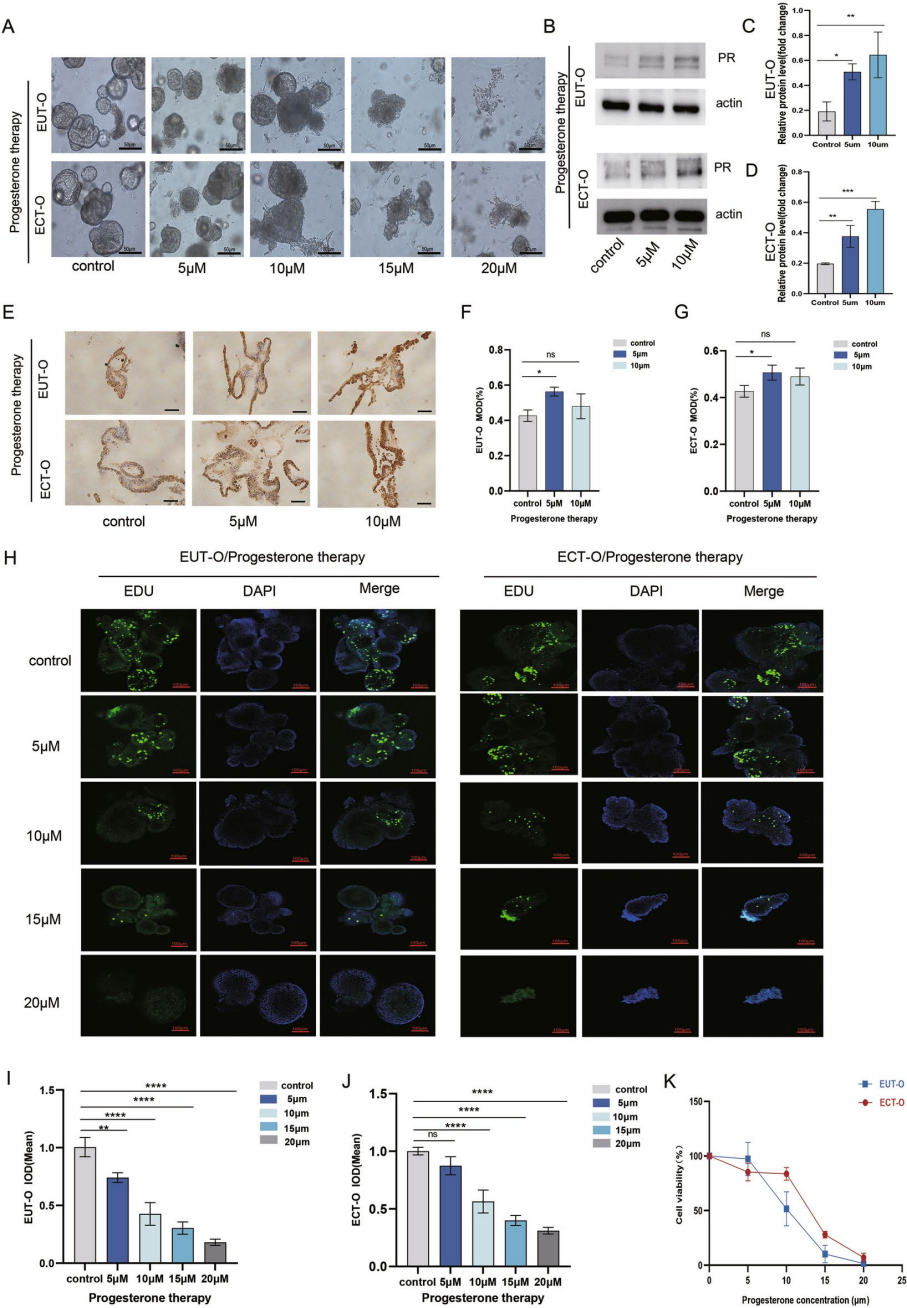
Morphological and Functional Changes in OE Endometrial Epithelial Organoids under Progesterone Intervention (**A**) High-magnification microscopy observation of EUT-O and ECT-O growth after 7 days of progesterone intervention (scale: 50 μm).(**B**) Western blot was used to detect the protein levels of PR in different groups:control , PR(5 μM), PR(10 μM). (**C**) Western blot was used to detect the protein levels of PR in EUT-O:control , PR(5 μM), PR(10 μM).(**D**) Western blot was used to detect the protein levels of PR in ECT-O:control , PR(5 μM), PR(10 μM). (**E**) Estrogen receptor expression in EUT-O and ECT-O under 5–10 μM progesterone intervention (scale: 20 μm) (**F**) Quantitative analysis of estrogen receptor expression in EUT-O under 5–10 μM progesterone intervention. (**G**) Quantitative analysis of estrogen receptor expression in ECT-O under 5–10 μM progesterone intervention. (**H**) Laser confocal images of EDU-labeled proliferating EUT-O and ECT-O under progesterone intervention (scale: 100 μm). (**I**) Immunofluorescence quantification of EDU-labeled proliferation in EUT-O under progesterone intervention. (**J**) Immunofluorescence quantification of EDU-labeled proliferation in ECT-O under progesterone intervention. (**K**) 3D viability assessment of ECT-O under different progesterone concentrations.

To further explore the responsiveness of endometrial epithelial organoids to progesterone, different concentrations of progesterone (5 μM, 10 μM, 15 μM, and 20 μM) were used to intervene with two types of organoids. Under high magnification microscopy, both types of organoids exhibited scattered individual cells around the glands. As progesterone concentration increased, the glands gradually ruptured and disintegrated, and most organoids even underwent apoptosis (Fig. 6A). Subsequently, surviving EUT-O and ECT-O organoids cultured with 5-10 μM progesterone were collected to assess PR expression (Fig. 6E). Notably, moderate progesterone concentrations led to a slight increase in PR expression in both EUT-O and ECT-O, with statistically significant up-regulation was observed at the 5 μM concentration (p < 0.05) (Fig. 6F,G). However,WB results verified at the protein level that progesterone at 5-10 μM concentration caused a slight increase in PR expression in EUT-O and ECT-O (p < 0.05) (Fig. 6B,C,D).Additionally, using laser confocal microscopy and a 3D viability assay, proliferative activity of OE organoids following different progesterone interventions was assessed. The fluorescence intensity of surviving OE organoids gradually decreased with increasing progesterone concentration (Fig. 6H). And semi-quantitative analysis indicated a significant decline in proliferative activity associated with higher progesterone concentrations (Fig. 6J,K). Furthermore, the 3D viability assay confirmed that excessive progesterone inhibited the activity of OE organoids (Fig. 6L).

## Discussion

Endometriosis is the most common gynecological benign tumor, and its pathogenesis is complex and remains unclear^16^. The main cell components in endometriosis lesions are epithelial cells and stromal cells^17^. It is not difficult to extract the stromal cells and there are many publications describe it while the viability of endometrial epithelium and passage is difficult so most of the endometriosis models are using stromal cells which is not enough to describe the pathogenesis and tissue homeostasis of endometriosis so the importance of 3D model ^18,19^. Typical organoids, a simple epithelial cell model, are expected to address this issue^15^. This study has successfully constructed 24 cases of eutopic endometrial epithelial organoid and 18 cases of ectopic endometrial epithelial organoid for OE, overcoming the research challenges of endometriosis and providing a reliable new model for further mechanistic studies of the disease.

In this study, we attempted to construct two types of endometrial epithelial organoids for OE. H&E staining revealed that the glandular arrangement in the eutopic endometrial epithelial organoids was relatively intact and morphologically regular, with visible glycogen secretion in the glandular lumens highly similar to the original tissue structure. The glandular cells in the ectopic endometrial epithelial organoids were disorganized and structurally atypical, with a few differentiated glandular lumens highly similar to the original histopathological structure. Ultrastructural analysis further confirmed the similarity between the cultured eutopic and ectopic endometrial epithelial organoid models and the parental tissue structure. Additionally, STR genetic kinship identification showed that the passaged organoids matched the original tissue perfectly, with no DNA anomalies, indicating that potential genetic biomarkers in the patient’s tissue were stably maintained in the corresponding organoids. The application of colorectal organoids in a study using a machine learning-based image analysis tool also indicated no significant morphological differences after passaging, consistent with our observations^16^. Microscopic observation showed that after multiple passages, there were no significant changes in the eutopic and ectopic endometrial epithelial organoid models.

Although microscopic observation revealed that both types of organoids possess cystic, mixed, solid, diffuse 3D structures, and the majority are predominantly cystic. However, during the culture process, it was found that most of the freshly obtained tissues, particularly the ectopic endometrial tissues with a harder texture and poor blood supply, were difficult to culture. In contrast, tissues with good blood supply and softer texture, both eutopic and ectopic endometrial tissues, were easier to culture and grew faster. Nevertheless, the construction time for most ectopic endometrial epithelial organoids was longer than that for eutopic endometrial epithelial organoids, with slower growth and a slightly lower success rate. This may be due to the fibrosis of the ectopic cyst wall in endometriosis, resulting in fewer glandular epithelial cells within the tissues, thus increasing the difficulty of culturing ectopic endometrial epithelial organoids^17^. H&E staining showed a considerable difference in the internal structure between the two types of organoids. Although ectopic endometrial epithelial organoids also had complete and orderly arranged glandular lumens forming circular structures, they were less in number compared to eutopic endometrial epithelial organoids. Moreover, the glandular cells in most ectopic endometrial epithelial organoids were more disorganized and sparse, with obvious structural atypia. Ultrastructural examination under an electron microscope indicated that the ectopic endometrial epithelial organoids were more severely damaged than the eutopic ones. This may also be one of the factors affecting the construction of ectopic endometrial epithelial organoids.

Previous studies have shown high levels of estrogen in endometriosis tissues. In this study, we attempted to add different concentration estrogen to the organoids. An appropriate amount of estrogen was found to promote the construction of OE epithelial organoid, with concentrations of 5-10 μM estrogen enhancing the growth of OE endometrial epithelial organoids, significantly improving their successful construction rate. Past research has indicated that progesterone can be one of the standard and effective methods for treating patients with endometriosis^18^. In this study, we further intervened organoids with progesterone drugs, and the OE endometrial epithelial organoids decreased with increasing concentrations of progesterone, accompanied by a decline in proliferative activity. These organoids were highly sensitive to progesterone drugs, which could inhibit the growth of ectopic endometrial epithelial organoid and lead to structural damage or even disappearance. Since the cultured ectopic endometrial epithelial organoids originate from human tissues, their sensitivity and response to estrogen and progesterone exhibit individual variations, making them a new model for future individual patient drug efficacy testing.

In previous research, endometrial stromal cells derived from the endometrium have been used as a cellular model for basic research on endometriosis. The genetic characteristics of traditional 2D cultured cell lines may change over multiple generations, which does not accurately reflect the heterogeneity, histopathology, and genetic characteristics of cells in vivo. However, the endometriosis organoids we have established could maintain structures and physiological properties similar to the original tissues which are also sensitive to estrogen and progesterone. In fact, organoids could serve as a reliable research model and have now been proven as a stable cell model in study of tumors^19–21^.

However, organoids derived from patients still exhibit certain limitations, primarily due to the absence of the surrounding microenvironment and various cell types, which affect the occurrence and development of lesions^22^. As we all know, the microenvironment and stromal cells play a crucial role in the pathogenesis and progression of diseases, with the peritoneal microenvironment influencing lesions in both pathogenic mechanism studies and clinical treatments. When establishing organoid models, the lack of a microenvironment within the lesions means that they can not fully simulate the critical influence of the local inflammatory microenvironment and surrounding immune cells, which are characterized by terminal heterogeneity^23–25^. Moreover, future explorations into the vascularization of organoid models remain significant, as the co-culture of immune cells with the stroma is still crucial for achieving precision in OE epithelial cell models.(Fig S1)

## Conclusion

In this study, we successfully established two types of ovarian endometriosis endometrial epithelial organoids and identified the different effects of hormones on the organoid models, which represent key phenotypic and functional features of endometrial tissue in endometriosis.

## Supplementary Information

Supplementary Information 1.

Supplementary Information 2.

Supplementary Information 3.

Supplementary Information 4.

Supplementary Information 5.

Supplementary Information 6.

## Data Availability

The data is available from the corresponding author on reasonable request.
