# Supplementary figures and images for: Construction organoid model of ovarian endometriosis and the function of estrogen and progesterone in the model
Source: Sci Rep. 2025 Feb 24;15:6636. doi: 10.1038/s41598-025-90329-0 (PMC11850836; doi:10.1038/s41598-025-90329-0)

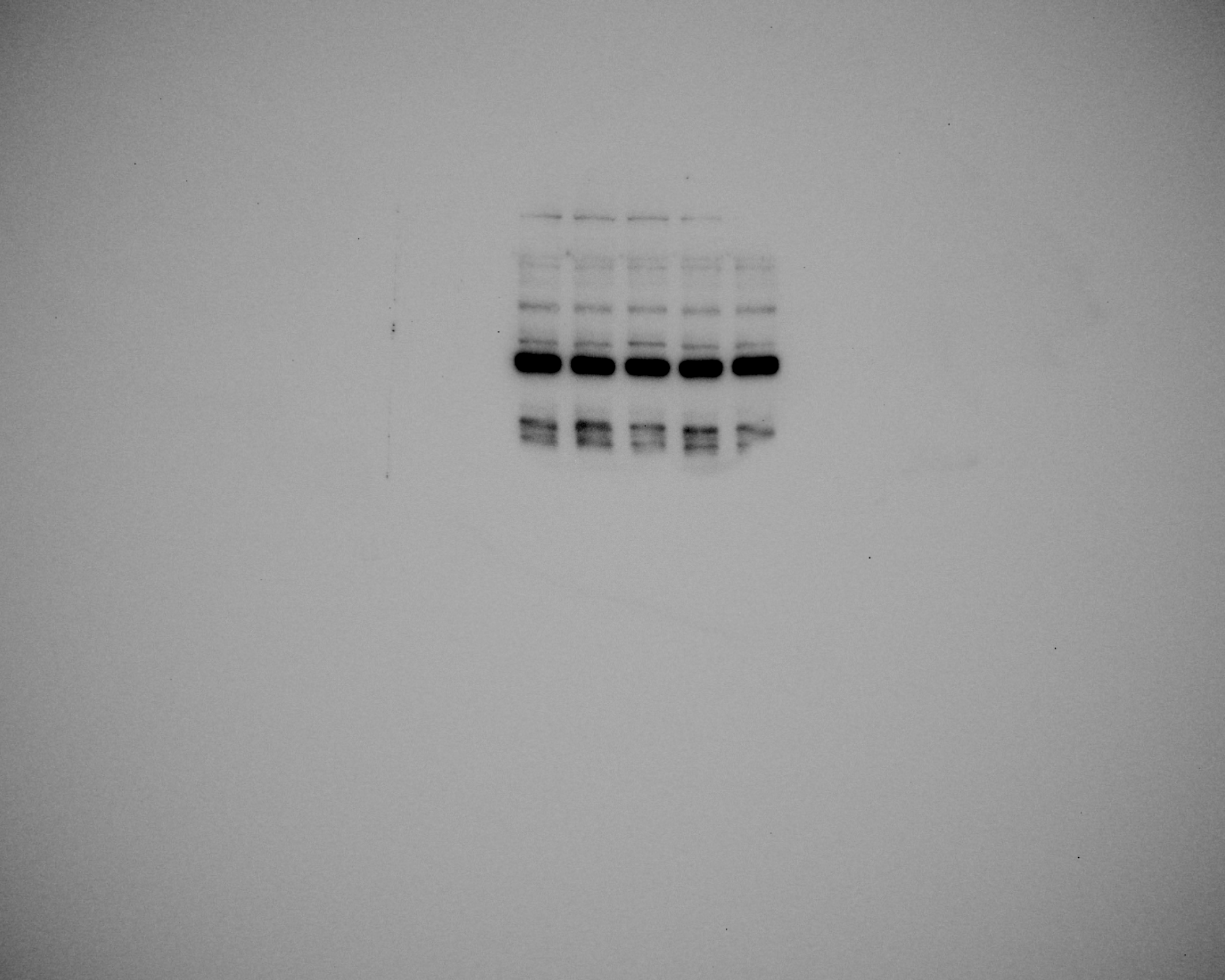

Supplement: Supplementary file 1 — Supplementary Information 1. [file 41598_2025_90329_MOESM1_ESM.tif]

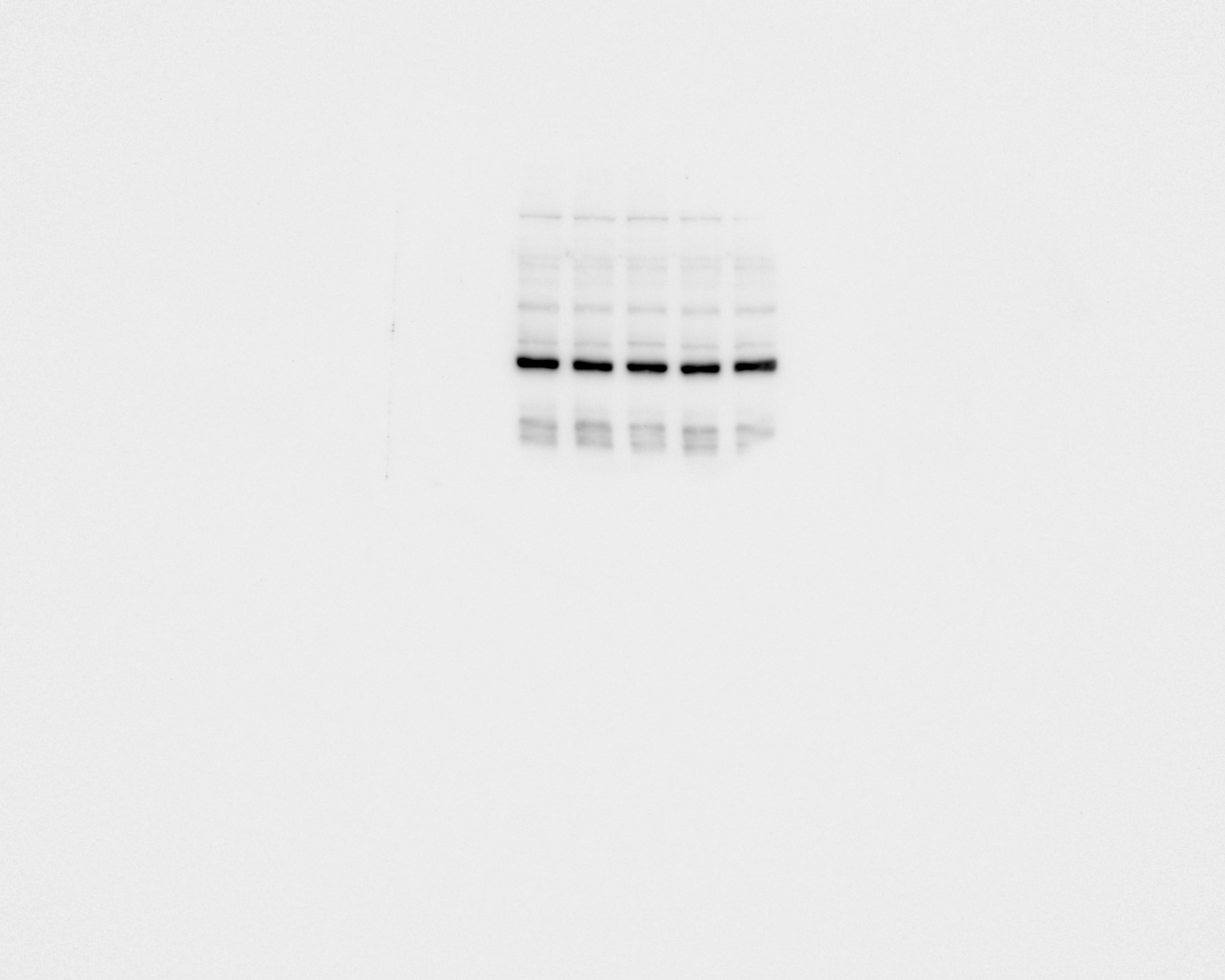

Supplement: Supplementary file 2 — Supplementary Information 2. [file 41598_2025_90329_MOESM2_ESM.tif]

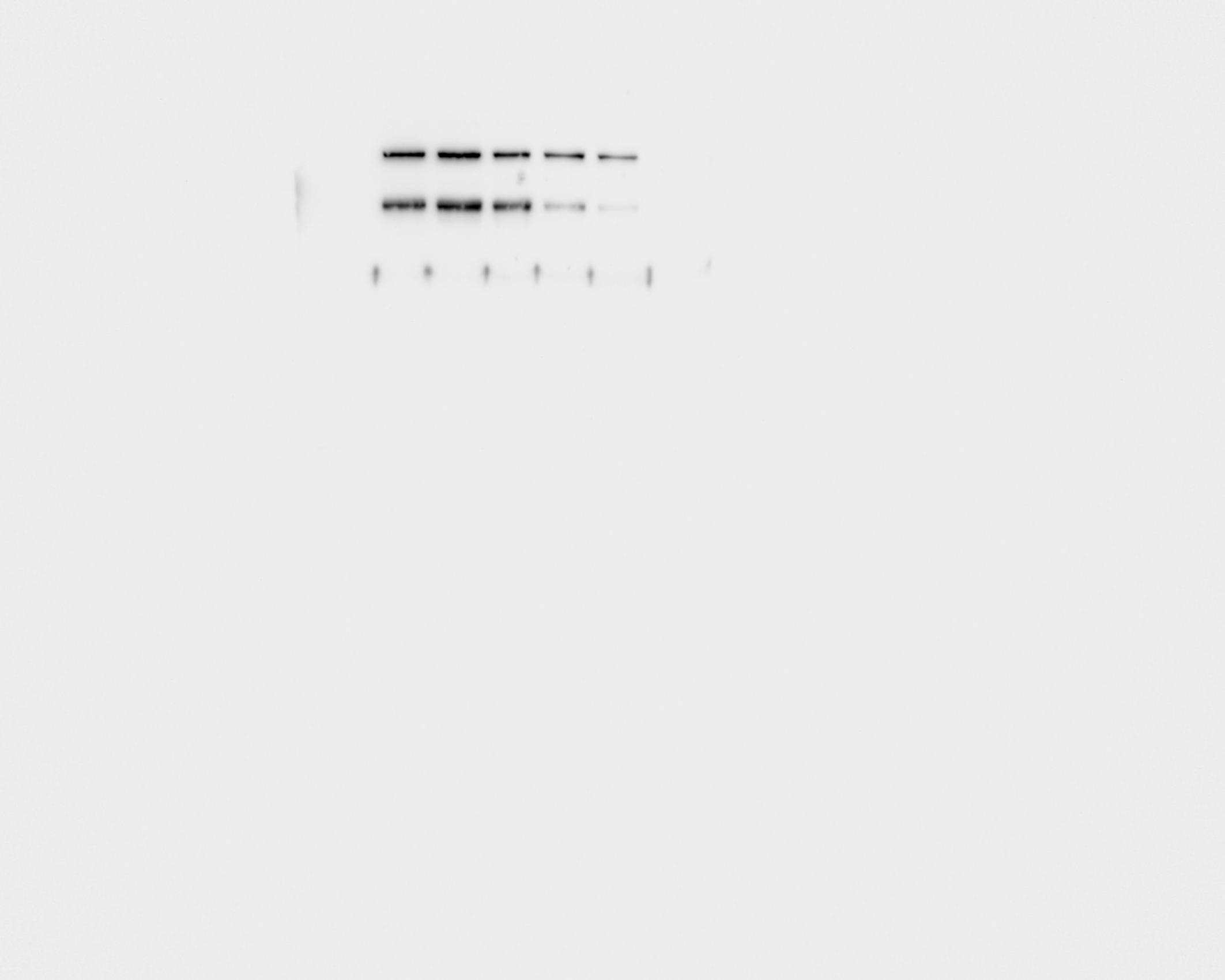

Supplement: Supplementary file 3 — Supplementary Information 3. [file 41598_2025_90329_MOESM3_ESM.tif]

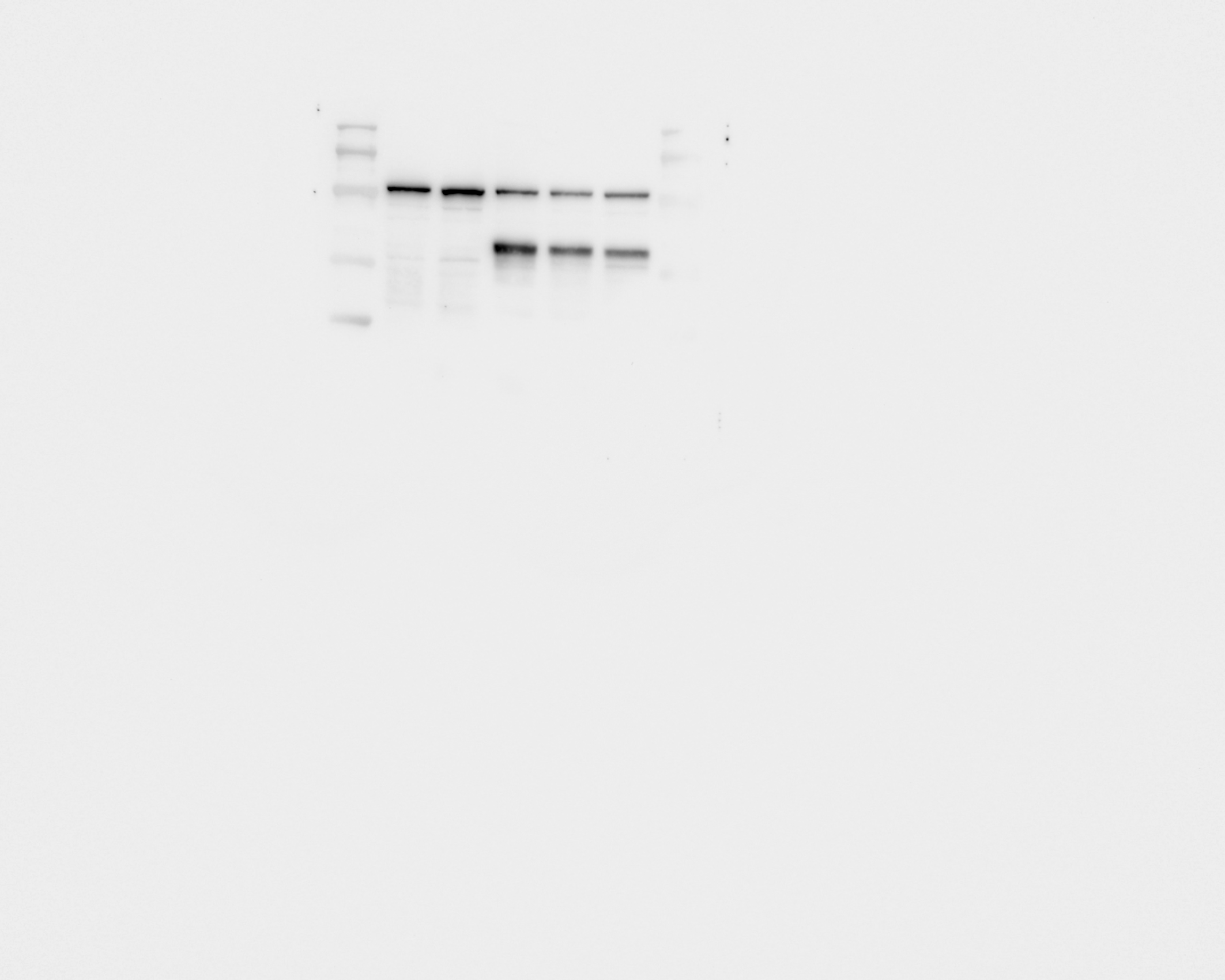

Supplement: Supplementary file 4 — Supplementary Information 4. [file 41598_2025_90329_MOESM4_ESM.tif]

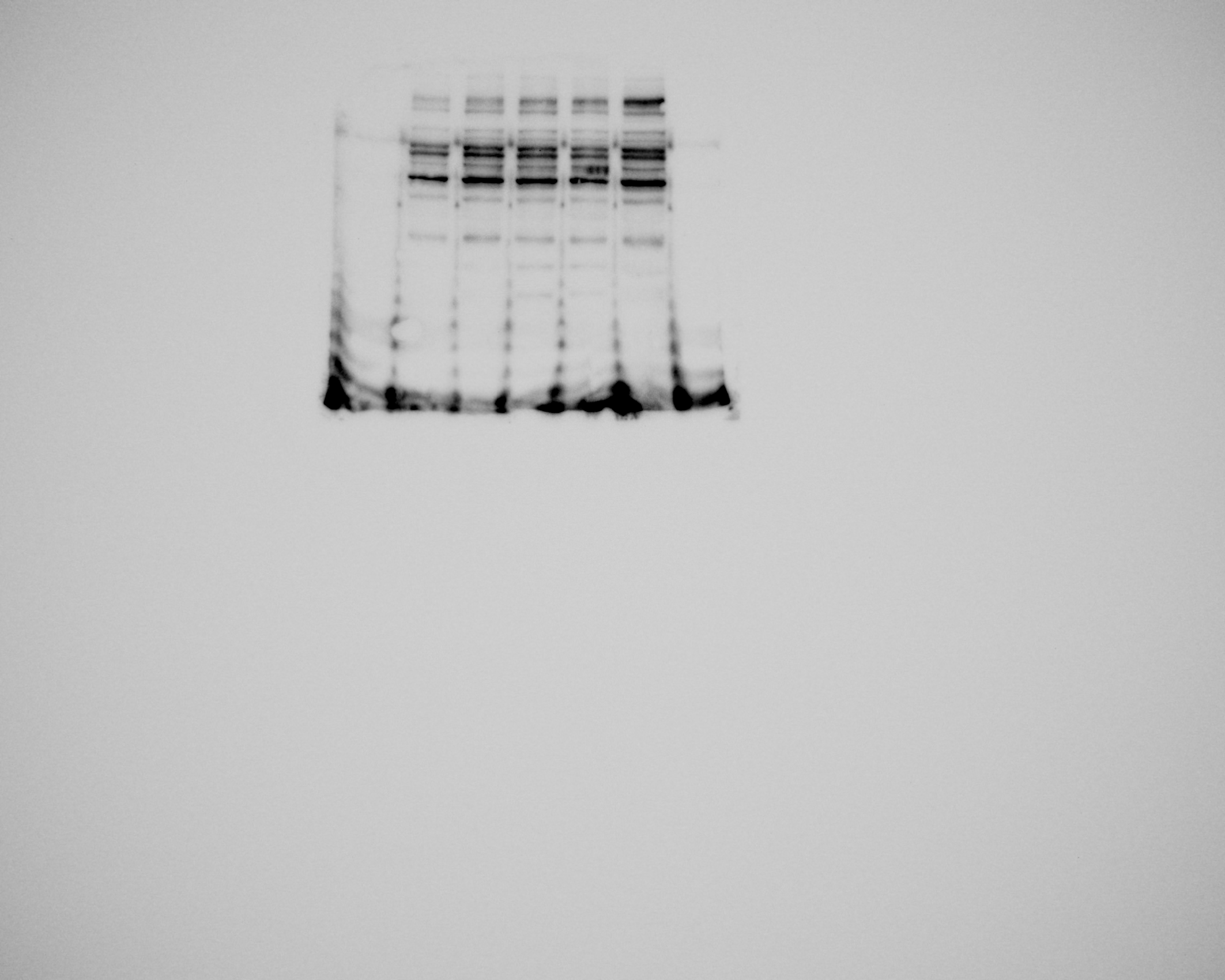

Supplement: Supplementary file 5 — Supplementary Information 5. [file 41598_2025_90329_MOESM5_ESM.tif]

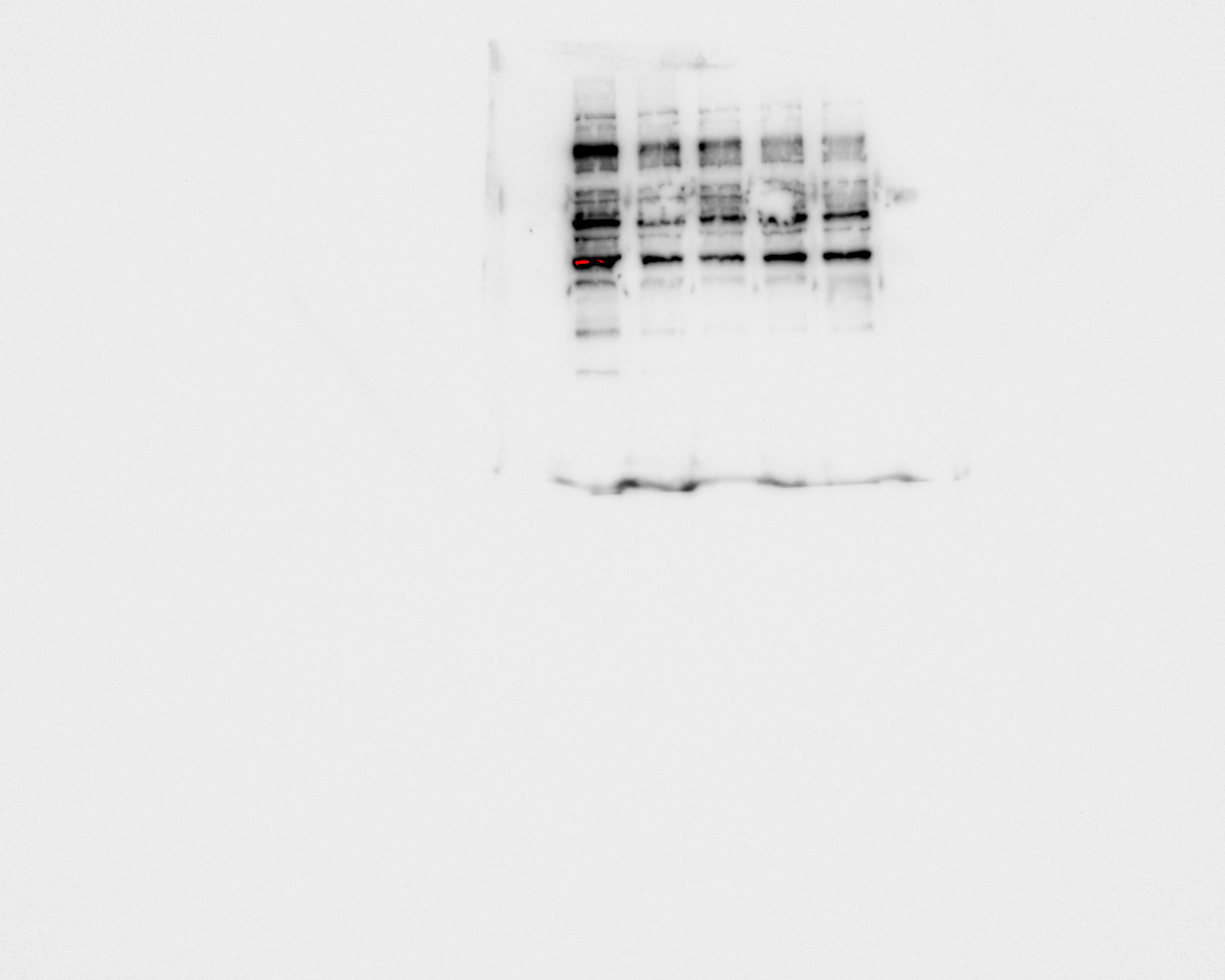

Supplement: Supplementary file 6 — Supplementary Information 6. [file 41598_2025_90329_MOESM6_ESM.tif]
